# Mitigating chronic respiratory disease through the lens of multimorbidity: the MARES mixed-methods study protocol

**DOI:** 10.1136/bmjopen-2025-109950

**Published:** 2026-01-16

**Authors:** Renata Gonçalves Mendes, Naiara Tais Leonardi, Viviane Castello-Simões, Débora Mayumi de Oliveira Kawakami, João Victor Rolim Souza, Nathany Souza Schafauser-Segundo, Rodrigo Polaquini Simões, Fernanda Gabriely Pinto, Gustavo Henrique Guimarães Araujo, Marcela Maria Carvalho da Silva, Cristiane Shinohara Moriguchi, Francisco José Barbosa Zörrer Franco, Valéria A Pires Di Lorenzo, Rachel Jordan, Sonia Martins, Trishul Siddharthan, Saleh Al Sharmah, Julie Barber, John R Hurst

**Affiliations:** 1 Physical Therapy Department, Federal University of São Carlos, São Carlos, Brazil; 2 University of Santo Amaro, São Paulo, Brazil; 3 Bioengineering Program, Scientific and Technological Institute, Brazil University, São Paulo, Brazil; 4 Rede D’Or Sao Luiz, São Paulo, Brazil; 5 Public Health and Epidemiology, University of Birmingham, Birmingham, UK; 6 Center for Public Health Studies (CESCO), University Center of the ABC Medical School, São Paulo, Brazil; 7 Department of Medicine, Division of Pulmonary, Critical Care and Sleep Medicine, University of Miami, Coral Gables, Florida, USA; 8 UCL Respiratory, University College London, London, UK; 9 Department of Statistical Science, University College London, London, UK

**Keywords:** Primary Health Care, Multimorbidity, Pulmonary Disease, Chronic Obstructive

## Abstract

**Introduction:**

Chronic respiratory diseases (CRDs), such as asthma and chronic obstructive pulmonary disease (COPD), are among the leading non-communicable diseases (NCDs) worldwide. However, diagnosing CRDs in low-income and middle-income countries (LMICs) remains challenging due to limited access to spirometry and trained professionals. Aggravating the burden, CRDs often coexist with other NCDs, increasing healthcare costs, reducing quality of life and elevating mortality. These challenges highlight the need for simple case-finding approaches for CRDs, such as the COPD in Low-Income and Middle-Income Countries Assessment (COLA-6) questionnaire, to support prompt identification and appropriate care within NCD services in LMICs.

**Objective:**

To evaluate the discriminative accuracy, feasibility and implementation of the COLA-6 questionnaire in identifying and managing CRDs in Brazilian Primary Healthcare (PHC) services for NCDs.

**Methods and analysis:**

The Multimorbidity Approach for REspiratory Solutions (MARES) study consists of three work packages to be conducted in PHC services in São Carlos/SP and São Paulo/SP, Brazil.

MARES-1: A cross-sectional observational study enrolling 859 individuals with at least one NCD receiving care in PHC. The COLA-6 questionnaire will be administered by the research team and compared with quality-assured spirometry. The Chronic Airways Assessment Test (CAAT), Asthma Control Questionnaire (ACQ-7) and fractional exhaled nitric oxide (FeNO) will also be assessed. The diagnostic performance of COLA-6 for identifying CRDs—including COPD, asthma, preserved ratio impaired spirometry, restriction and overlaps—will be assessed using area under receiver operating characteristic curves and 95% CIs.

MARES-2: A cross-sectional observational study enrolling 20 healthcare professionals (physicians, physiotherapists, community health agents and nurses) from five PHC services. These professionals will apply the COLA-6 during routine NCD care to a total sample of 1000 patients. Qualitative interviews will be conducted to explore barriers and facilitators to the implementation of COLA-6, using deductive thematic analysis.

MARES-3: A longitudinal, prospective observational study in which patients from MARES-1 and MARES-2 will be reassessed at 6-month follow-up. A total sample of 473 participants with abnormal spirometry, a diagnosis of CRD or high risk for CRDs is expected. Participants will undergo spirometry, and a subset will be interviewed to explore their healthcare experiences through qualitative thematic analysis. Access to diagnostic and treatment services in Brazil will be assessed. Changes in spirometry values, FeNO, CAAT and ACQ-7 scores from baseline to 6 months in patients from MARES-1 will be analysed.

**Ethics and dissemination:**

This study has been approved by the Ethics Committees of Federal University of São Carlos and University of Santo Amaro (UNISA). Ethical approval was also granted by the University College London. Results will be disseminated through peer-reviewed medical journals and presentations at international conferences. Results will improve identification of CRDs, addressing a significant gap in current PHC settings.

**Trial registration number:**

NCT07050823/NCT07093021/NCT07134855.

**STRENGTHS AND LIMITATIONS OF THIS STUDY**

- Considers chronic respiratory disease (CRD) diagnoses including asthma and chronic obstructive pulmonary (COPD) disease together and in the setting of established care for non-communicable diseases.
- Assesses the real-world feasibility of implementing the COPD in Low-Income and Middle-Income Countries Assessment questionnaire (COLA-6) among healthcare professionals and patients in primary healthcare (PHC) settings.
- Multiphase, mixed-methods approach combining cross-sectional and longitudinal designs with both quantitative and qualitative analyses.
- Findings may be specific to Brazil’s PHC system and may not be directly generalisable to other healthcare settings or regions in low-income and middle-income countries.
- A 6-month follow-up may not fully capture long-term outcomes related to improved CRD diagnosis and management.

## Introduction

 Chronic respiratory diseases (CRDs) are among the most prevalent chronic non-communicable diseases (NCDs) worldwide.^1^ Asthma and chronic obstructive pulmonary disease (COPD) are the most common CRDs.^2^ The global prevalence of asthma was 262 million people in 2019, causing 455 000 deaths, while COPD was responsible for 480 million cases in 2020 and approximately 3 million deaths in 2019.^2^,^5^ High-income countries report higher COPD prevalence than low-income and middle-income countries (LMICs), partly due to age distribution differences and underdiagnosis. However, rising life expectancy in LMICs may lead to an increased prevalence.^6^ The combined prevalence of asthma and COPD exceeds that of diabetes (463 million) or HIV infection (39 million).^7 8^

To confirm COPD and asthma in individuals presenting with characteristic symptoms, lung function must be evaluated, most often through spirometry, a test that remains scarce in LMICs, including Brazil, where the majority of people with CRDs live.^9^,^14^ Spirometry requires specific equipment, resources for implementation within health systems, trained professionals to perform the exam and clinicians capable of interpreting the results.^12^ Consequently, many patients with CRDs are either not diagnosed or are diagnosed late in the course of the disease. Undiagnosed and consequently untreated patients make significant use of healthcare services and are at increased risk of disability, acute exacerbations and premature death.^15^ When diagnosed early, with greater treatment potential, a lower disease burden and a better long-term prognosis become possible.^16^ This aligns with the recent resolution of the 78th World Health Assembly, which calls for strengthening primary healthcare (PHC) through integrated strategies, recognising shared risk factors among lung and other NCDs, and promoting evidence-based, cost-effective approaches for early detection and treatment.^17^

Prompt identification of CRDs is therefore crucial not only for those affected but also for the health system and society as a whole. Given the previously described barriers to CRD diagnosis, alternative approaches to conventional spirometry for detecting undiagnosed and untreated cases are required to enable earlier therapeutic management, reduce symptoms, and the frequency and severity of respiratory infections (exacerbations), and ultimately improve quality of life and survival.^18^

As a strategy for identifying individuals at risk of CRDs in LMICs, particularly COPD, Siddharthan *et al*^19^ developed and validated a simple ‘case-finding’ tool comprising a seven-item questionnaire combined with a simple measurement of lung function (peak expiratory flow or PEF): the ‘COPD in Low-Income and Middle-Income Countries Assessment’ (COLA). The ‘Global Excellence in COPD Outcomes’ (GECo) group demonstrated the discriminative accuracy of the tool in identifying individuals with COPD in a sample of 10 664 people from three LMICs (Nepal, Peru and Uganda). In that study, COLA was applied in a short version (COLA-6) and found to be feasible and accurate.^19^ However, the authors emphasised the need to test the performance of COLA-6 in other LMICs and to determine whether its implementation by clinicians rather than research staff is associated with improved clinical outcomes. The COLA-6 questionnaire has since been translated and cross-culturally adapted into Brazilian Portuguese for use in Brazil.^20^ In general, previous studies have considered asthma and COPD separately, yet in a PHC setting, it may be more cost-effective to consider CRDs together. Indeed, there is a third, prevalent pattern of spirometric abnormality termed PRISm (preserved ratio impaired spirometry) that is also associated with significant respiratory morbidity and mortality.^21^

In parallel with the burden of CRDs, there has been growing interest in and concern about multimorbidity, defined as the coexistence of at least two long-term conditions in the same individual.^22 23^ CRDs frequently coexist with other NCDs, and this coexistence has been associated with increased hospital admissions and healthcare costs, as well as reduced quality of life and exercise tolerance.^24^ Furthermore, the risk of comorbidities is higher in patients with COPD compared with matched controls, and these comorbidities strongly influence mortality.^25^ Case-finding for undiagnosed COPD in people with hypertension appears feasible in PHC in Brazil,^26^ but the benefit to individuals and society remains to be defined. Multimorbidity in patients with asthma can contribute to an increase in symptoms and worsening control of the disease.^27^ Although the mechanisms underlying the relationship between CRDs and other chronic diseases are not yet fully understood, several have been proposed, including shared environmental exposures (eg, tobacco use, air pollution, biomass smoke), physical inactivity (driven by dynamic hyperinflation, muscle wasting) and accelerated ageing (cellular senescence, systemic inflammation, oxidative stress).^24^

In summary, a more comprehensive approach to individuals with CRDs—considered collectively—and multimorbidity is essential for the development of multidimensional assessment and holistic care strategies aimed at improving patient outcomes^28^ and reducing health service burden. Thus, considering the complexity of these patients, and through the lens of multimorbidity, there is a strong clinical and public health rationale for identifying people living with undiagnosed CRDs during routine care for other NCDs in PHC services. This would be of particular value in LMICs, including Brazil. However, despite its potential benefits, it remains unknown whether case-finding approaches using simple tools like COLA-6 can be effectively implemented during routine NCD care and what the short-term and longer-term benefits may be for both patients and the broader healthcare system.

### Objective

*Multimorbidity Approach for* *REspiratory Solutions (MARES-1)*: To evaluate the discriminative accuracy of the COLA-6 questionnaire in identifying Brazilian individuals with CRDs who attend PHC services for the management of other NCDs.

*MARES-2*: To assess the feasibility of implementing the COLA-6 questionnaire in PHC settings for NCD care in Brazil by the PHC professionals.

*MARES-3*: To investigate how individuals with NCDs attending PHC services, identified as having abnormal spirometry, CRD or high risk for CRDs, access further diagnostic and treatment services in Brazil.

## Methods and analysis

### Study design overview

This study is delivered as an equitable international partnership between University College London (UCL), UK, and the Federal University of São Carlos (UFSCar), Brazil. The project spans 4 years, beginning in 2025 and planned to conclude in 2028, and it is entitled MARES—Multimorbidity Approach for REspiratory Solutions. Ethical approval has been granted by the Human Research Ethics Committee of UFSCar and National Research Ethics Commission and the University of Santo Amaro (UNISA) (reference number: MARES-1: 85805425.4.1001.5504; MARES-2: 85837225.7.1001.5504; MARES-3: 85813325.3.1001.5504), a collaborating partner institution of the Basic Health Unit (BHU) in São Paulo/SP. Ethical approval was also granted by UCL Research Ethics (reference: 2024/0048). All participants will have their identities and personal data treated with strict confidentiality. Participation will only take place after signing the Informed Consent Form, corresponding to the specific study in which they are enrolled. The studies have been registered at clinicaltrials.gov as MARES-1 (NCT07050823), MARES-2 (NCT07093021) and MARES-3 (NCT07134855).

*MARES-1*: This study aims to evaluate the discriminative capacity of the COLA-6 questionnaire in identifying individuals with CRDs who attend PHC services in Brazil for the treatment of other NCDs. To achieve this, we will conduct a cross-sectional observational study enrolling 859 individuals with one or more existing NCDs who are receiving care in PHC services. The COLA-6 questionnaire will be administered, and its results will be compared with gold-standard postbronchodilator spirometry. The performance of the case-finding tool will be assessed using the area under a receiver operating characteristic (ROC) curve for CRDs considered collectively and separately.

*MARES-2*: This study aims to evaluate whether the COLA-6 questionnaire is feasible to be implemented by health professionals during routine NCD care in PHC services in Brazil. For this purpose, we will conduct a cross-sectional observational study enrolling 20 healthcare professionals (physicians, physiotherapists, community health agents and nurses) from five PHC services to apply the COLA-6 questionnaire during routine NCD care. This is expected to result in a total sample of 1000 patients. Qualitative interviews will be conducted with the healthcare professionals to explore barriers and facilitators to implementing the COLA-6 in clinical practice.

*MARES-3*: This study aims to investigate how individuals attending PHC services in Brazil for NCD treatment, who are either identified as having abnormal spirometry or a diagnosis of CRD in MARES-1 or identified as being at high risk for CRDs through the COLA-6 questionnaire in MARES-2, access further diagnostic and treatment services. To this end, we will conduct a longitudinal, prospective observational study. This follow-up study, at 6 months, will include an estimated sample of 309 individuals with abnormal spirometry results or diagnosis of CRDs from MARES-1 and an estimated sample of 164 individuals identified as being at high risk for CRDs through the COLA-6 questionnaire in MARES-2, resulting in an estimated total sample of 473 participants. Qualitative interviews will be conducted with a diverse subsample of 30 participants, to explore the barriers and facilitators to accessing the healthcare system for diagnosis and treatment, while all participants will undergo basic data collection during follow-up.

### Study setting

This study will be conducted in PHC services, including BHUs and Family Health Units (FHUs), located in the municipalities of São Carlos and São Paulo, both situated in the state of São Paulo, Brazil. The municipality of São Carlos has a population of 254 857 inhabitants and a gross domestic product (GDP) of R$55 044.88 per capita,^29^ with 34 PHC units.^30^ In contrast, São Paulo, the state capital, has a population of 11 451 999 inhabitants and a per capita GDP of R$66 872.84, and is served by 471 PHC units.^29 31^ A total of 35 BHUs/FHUs will be involved in the study: 34 located in São Carlos/SP and one in São Paulo/SP (Jardim Cliper), in the southern region of the city.

### Participants and outcomes

This study will include two main groups of participants: (1) individuals with one or more NCDs who receive care from PHC services and (2) healthcare professionals working in these settings who provide care to such individuals. The eligibility criteria for each group, as they relate to MARES-1, MARES-2 and MARES-3 are detailed in table 1. The primary outcome for each objective, along with the instruments and measures used for their assessment, is presented in table 2.

**Table 1:** Eligibility criteria

Inclusion criteria	Exclusion criteria
MARES-1 and MARES-2	
Individuals	
Attending PHC services Diagnosed with one or more NCDs Aged ≥30 years	Clinically unstable in the past month (worsening symptoms requiring emergency care or hospitalisation) Currently pregnant Active pulmonary tuberculosis or undergoing treatment for pulmonary tuberculosis Acute respiratory infection Cognitive impairment that prevents understanding of the COLA-6 questionnaire, that is, MMSE score ≤20^48^ Contraindications to spirometry or inability to perform the test Declining to participate in the study

MARES-2	
Healthcare professional	
Providing care to individuals with NCDs attending PHC services Includes physicians, nurses, physiotherapists and community agents	Did not complete training to apply the COLA-6 questionnaire Declining to participate in the study

MARES-3	
Individuals	
The inclusion and exclusion criteria will be the same as those for individuals of MARES-1 and MARES-2, considering that this is a follow-up from these previous studies.	
Participants will be included in the follow-up phase if they meet either of the following criteria: Identified as having abnormal spirometry or diagnosis of CRD in MARES-1 Identified as being at high risk for CRD through the COLA-6 questionnaire in MARES-2	Participants will be excluded from the follow-up phase if: Clinically unstable in the past month (worsening symptoms requiring emergency care or hospitalisation) Currently pregnant Diagnosed with active pulmonary tuberculosis or undergoing treatment for pulmonary tuberculosis Presenting with an acute respiratory infection Having contraindications to spirometry or inability to perform the test Declining to participate in the study

COLA-6, short version of COPD in Low-Income and Middle-Income Countries Assessment; COPD, chronic obstructive pulmonary disease; CRD, chronic respiratory disease; FeNO, fractional exhaled nitric oxide; MARES, Multimorbidity Approach for REspiratory Solutions; MMSE, Mini–Mental State Examination; NCDs, non-communicable diseases; PHC, primary healthcare.

**Table 2:** Relationship between objectives, outcomes and measures

Objectives	Outcomes	Measures
MARES-1		
To evaluate the discriminative accuracy of the COLA-6 questionnaire in identifying individuals at risk for CRDs who attend PHC services for the treatment of NCDs	Discriminative accuracy of the COLA-6 questionnaire: Area under the receiver operating characteristics curve Sensitivity and specificity of the COLA-6 questionnaire with and without PEF	COLA-6 questionnaire Spirometry
	Correlation between COLA-6 questionnaire and SBQ questionnaire	COLA-6 questionnaire SBQ questionnaire

MARES-2		
To assess the feasibility of implementing the COLA-6 questionnaire in PHC settings for NCDs care in Brazil by primary care professionals	The barriers and facilitators to implementing the COLA-6 questionnaire	Semistructured interviews with health professionals

MARES-3		
To investigate whether individuals with NCDs, who use PHC services, identified as having abnormal spirometry or diagnosis of CRD or as being at high risk for CRDs, are able to access further diagnostic and treatment services	The barriers and facilitators to accessing the healthcare system for diagnosis and treatment	Semistructured interviews with patients
Mean change in inflammation of the airways		FeNO measurement
Mean change in lung function		Spirometry
Mean change in health status		CAAT questionnaire
Mean change in asthma control		ACQ-7 questionnaire

ACQ-7, Asthma Control Questionnaire; CAAT, Chronic Airways Assessment Test; COLA-6, short version of COPD in Low-income and Middle-income Countries Assessment; COPD, chronic obstructive pulmonary disease; CRDs, chronic respiratory diseases; FeNO, fractional exhaled nitric oxide; MARES, Multimorbidity Approach for REspiratory Solutions; NCDs, non-communicable diseases; PEF, peak expiratory flow; PHC, primary healthcare; PRISm, Preserved Ratio Impaired Spirometry; SBQ, Symptom-Based Questionnaire.

### Recruitment

For MARES-1, individuals will be recruited from PHC services, including BHUs and FHUs, located in the municipalities of São Carlos and São Paulo, Brazil. Eligible individuals with NCDs will be recruited through three primary strategies: (1) individuals receiving routine care for established NCDs at BHUs/FHUs, such as scheduled consultations for hypertension or diabetes; (2) individuals with NCDs identified through proactive outreach by community health agents and (3) individuals from BHUs/FHUs with NCDs who become aware of the study via passive recruitment strategies, including informational posters, communication from healthcare staff, peer referrals or invitations extended during service delivery days. Recruitment materials, including posters with contact details for the research team, will be displayed in BHUs/FHUs, and direct invitations will be issued during routine service encounters. All recruitment procedures will be conducted in accordance with ethical principles, ensuring the protection of participants’ rights to privacy and autonomy.

### Data collection, measures, management and analysis

**Figure 1:**
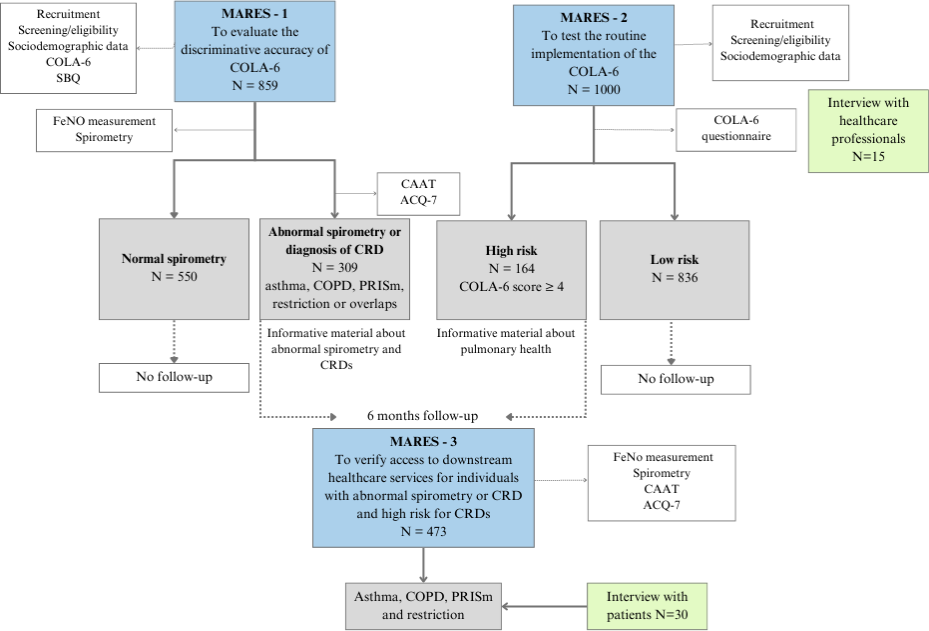
Overview of MARES-1, 2 and 3, with participant flow and assessments. ACQ-7, Asthma Control Questionnaire; CAAT, Chronic Airway Assessment Test; COLA-6, short version of COPD in Low-income and Middle-income Countries Assessment; COPD, chronic obstructive pulmonary disease; CRDs, chronic respiratory diseases; FeNO, fractional exhaled nitric oxide; MARES, Multimorbidity Approach for REspiratory Solutions; PRISm, Preserved Ratio Impaired Spirometry; SBQ, Symptom-Based Questionnaire.

Data collection will follow the study design and the patient flow described in figure 1.

#### MARES-1

Data collection will be conducted at a single visit by a member of the UFSCar research team. All eligible participants will provide written informed consent prior to study procedures. The following procedures will be carried out:

1. Initial assessment (details below).
2. Application of case-finding tools questionnaires:
  - COLA-6 questionnaire.^19 20^
  - Symptom-Based questionnaire (SBQ).^32^
3. Fractional exhaled nitric oxide (FeNO) measurement.
4. Spirometry (prebronchodilator and postbronchodilator).
5. Application of disease-specific questionnaires:
  - Chronic Airways Assessment Test (CAAT) for all individuals with abnormal spirometry or CRD.^33 34^
  - Asthma Control Questionnaire (ACQ-7) for individuals with isolated or overlapping asthma.^35 36^
6. Participants identified with abnormal spirometry results and diagnosis of CRD will be provided with an informational leaflet containing basic guidance about abnormal spirometry or asthma or COPD and how to access diagnostic and treatment services for respiratory conditions.

#### MARES-2

This study comprises three phases.

##### Phase 1: Implementation of COLA-6

Application of COLA-6 questionnaire: After receiving prior training from the UFSCar research team, healthcare professionals at the BHUs and FHUs will identify potentially eligible individuals with NCDs who attend PHC services and will administer the COLA-6 questionnaire as part of their routine care. Participants identified as high risk for CRDs based on COLA-6 results will be provided with an informational leaflet containing basic guidance about pulmonary health and how to access diagnostic and treatment services for respiratory conditions.

Fidelity monitoring: The research team at the UFSCar will observe the administration of the COLA-6 at the beginning and end of all applications by each healthcare professional to ensure adherence to the protocol.

##### Phase 2: Participant enrolment and assessment

Following the administration of the COLA-6 as part of routine care, healthcare professionals will inform individuals who meet the inclusion criteria about the research and invite them to participate. Individuals expressing interest will be referred to the research team, who will subsequently contact them to provide detailed study information and obtain written informed consent. On consent, the research team will conduct the initial assessment (details below).

##### Phase 3: Evaluation of implementation

On completion of the 50 COLA-6 applications, healthcare professionals will participate in qualitative interviews conducted by the UFSCar team to explore the barriers and facilitators to implementation in routine PHC settings.

### MARES-3

Participants with abnormal spirometry or diagnosis of CRD from MARES-1 and those identified as high risk for CRDs in MARES-2 will be contacted for a follow-up visit 6 months after their initial participation. Follow-up will take place at the respective BHUs/FHUs and will be conducted by the UFSCar research team in a single day. Multiple contact options will be collected at baseline, and up to three telephone attempts will be made at follow-up; participants lost to follow-up will be documented and analysed. Procedures include:

1. FeNO measurement.
2. Spirometry (prebronchodilator and postbronchodilator).
3. Application of disease-specific questionnaires:
  - CAAT for all individuals with abnormal spirometry or CRD.^34^
  - ACQ-7 for individuals with isolated or overlapping asthma.^36^
4. Basic information about access to health services for diagnosis and treatment will be provided to participants with abnormal spirometry and diagnosis of CRD.
5. Qualitative interviews with individuals selected to reflect diversity of participants (n=30) to explore their healthcare experiences, particularly regarding access to respiratory diagnosis and care.

### Assessments

All assessments will be conducted by a team of clinical researchers who were previously trained to ensure the accuracy and consistency of data collection.

MARES-1 initial assessment: The research team will collect information about medical history, CRD risk factors, history of infections, current medications and previous hospitalisations. Additional information on childhood growth and development, socioeconomic profile and anthropometric data will be gathered.

COLA-6 questionnaire: The COLA-6 questionnaire,^19^ translated and culturally adapted into Portuguese^20^ (see online supplemental material), will be administered (MARES-1 and MARES-2). The questionnaire comprises three sections: (1) six questions addressing respiratory symptoms, functional status and exposure to risk factors; (2) one question on age and (3) one question involving peak expiratory flow (PEF) measurement. Scoring assigns 1 point for each affirmative response to the six questions, 1 point if participant is aged ≥55 years, 1 point if PEF is between 250–399 L/min, and 2 points if PEF is <250 L/min, resulting in a total score ranging from 0 to 9 points. PEF will be measured using a Mini-Wright Standard Peak Flow Meter (Clement Clarke, Essex, England). Participants will perform three forced exhalations from full lung capacity, using a nose clip, and following proper technique (avoiding neck flexion and tongue obstruction of the mouthpiece). Standardised verbal encouragement will be provided, with a 30 s pause between manoeuvres. If the two highest values differ by more than 40 L/min, up to two additional manoeuvres will be performed, to a maximum of five attempts. The highest recorded PEF value will be used.^37^ A COLA-6 score ≥4 is considered a positive test, representing the optimal combination of sensitivity and specificity for predicting the risk of COPD.^19^ Participants identified as at risk of CRDs in MARES-2 will receive their results along with educational material developed with the support from our patient and public involvement (PPI) group. The materials provide information on CRDs, risk factors, symptoms, diagnosis and treatment.

SBQ: The SBQ questionnaire^32^ will be administered to compare performance with COLA-6 (MARES-1). The SBQ consists of 11 questions covering age, smoking history, body mass index, cough, wheezing, allergies, dyspnoea, exposure to dust or chemical particles and the presence of respiratory diseases during childhood. A total score of ≥17 indicates a higher risk for COPD.^32^

FeNO measurement: FeNO will be measured using a portable analyser (FeNObreathe, Geratherm Medical Latin America, Brazil), following American Thoracic Society guidelines^38^ to assess type 2 airway inflammation (MARES-1 and MARES-3). Subjects will be seated comfortably and instructed to inhale through the mouthpiece for 2–3 s to total lung capacity, followed by a steady exhalation lasting at least 6 s, without air leakage. An expiratory flow rate of 50 mL/s will be maintained, monitored by a sound signal. Up to six manoeuvres will be allowed, with 30 s intervals between attempts. The highest value among three acceptable measurements will be used, ensuring quality control by limiting deviation to ≤2.5 ppb or 10% within 15 min. FeNO levels will be classified as low/normal (<25 ppb), intermediate (25–50 ppb) or high (>50 ppb).^39^

Spirometry: Lung function will be assessed using a portable spirometer (Spirobank II Advanced, MIR, USA) to confirm or exclude CRDs (MARES-1 and MARES-3). Testing will follow American Thoracic Society and European Respiratory Society standards, ensuring A or B quality grades. ^40 41^At least three forced, acceptable and reproducible manoeuvres will be performed before and 20 min after inhalation of 400 mcg of salbutamol sulfate. Spirometry results will be reviewed and interpreted by a board-certified pulmonologist.

**Figure 2:**
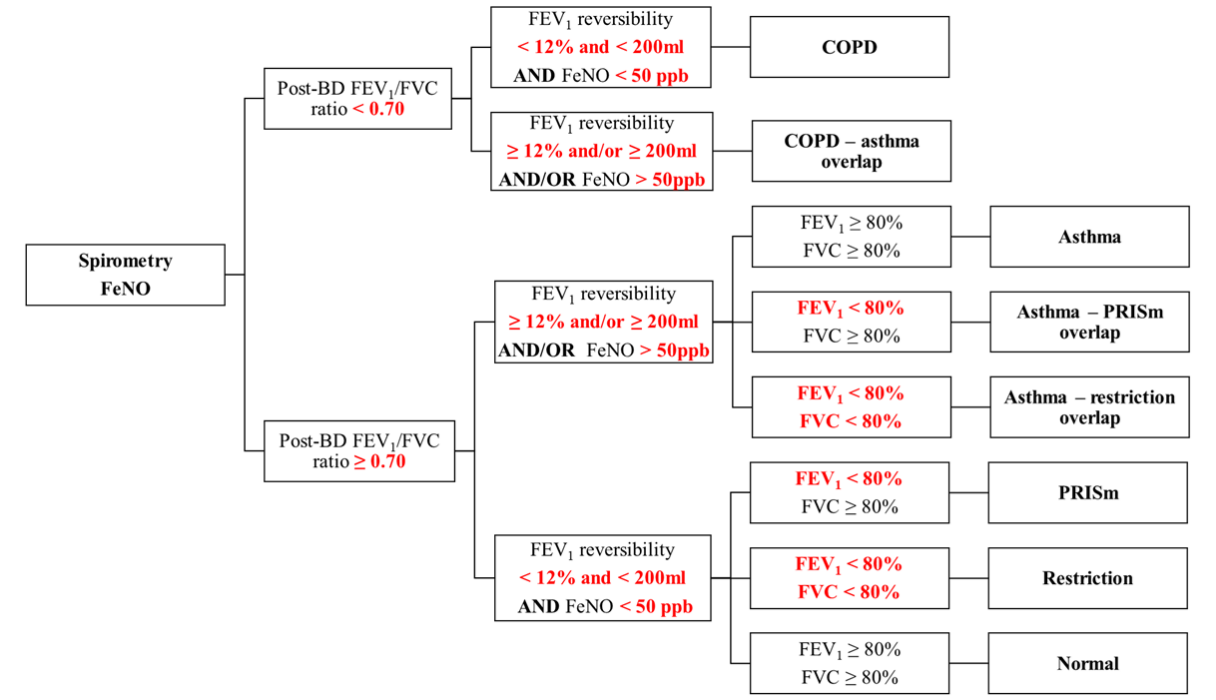
Classification using spirometry and FeNO measurement. BD, bronchodilator; COPD, chronic obstructive pulmonary disease; FeNO, fractional exhaled nitric oxide; FEV₁, forced expiratory volume in the first second; FVC, forced vital capacity; PRISm, Preserved Ratio Impaired Spirometry.

Participants will be classified based on spirometry and FeNO results (figure 2) into two groups, primarily according to the recommendations of the Global Initiative for Chronic Obstructive Lung Disease^42^ and the Global Initiative for Asthma.^27^

1. Abnormal spirometry or diagnosis of CRD includes COPD, asthma, asthma-COPD overlap, PRISm, restriction patterns, asthma-PRISm overlap and asthma-restriction overlap.
2. Normal spirometry: Participants without lung function abnormalities.

Those with abnormal spirometry results or a diagnosis of CRD in MARES-1 will receive their results, interpreted by a local pulmonologist using local Pereira reference equations^43^ along with educational materials co-developed with support from our PPI group, providing guidance on diagnosis, risk factors, symptoms and recommendations to seek appropriate healthcare services. For participants with asthma overlapping COPD, restrictive patterns or PRISm, asthma will be considered the primary condition for the purposes of health guidance and analysis.

CAAT and ACQ-7 questionnaires: After FeNO measurement and spirometry (MARES-1 and MARES-3), all individuals with abnormal spirometry or diagnosis of CRD will complete the CAAT questionnaire and will be asked about their history of previous exacerbations. Individuals diagnosed with asthma (isolated or overlapping with other conditions) will additionally complete the ACQ-7 questionnaire (MARES-1 and MARES-3). Both questionnaires have been validated for use in the Brazilian population.^34 36^ The CAAT questionnaire is used to assess the impact of CRD symptoms on daily life. It consists of eight items addressing cough, phlegm, chest tightness, dyspnoea when walking up a hill or one flight of stairs, limitations in daily activities, confidence in leaving the house, sleep quality and energy levels. Each item is scored from 0 to 5, with individuals selecting one answer per item. The total score (maximum of 40 points) indicates the clinical impact of the disease: 6–10 points: mild; 11–20 points: moderate; 21–30 points: severe; and 31–40 points: very severe.^34^ The ACQ-7 questionnaire assesses asthma control over the previous week. It includes six questions on asthma symptoms and short-acting bronchodilator use (each scored from 0 to 6) plus a measure of prebronchodilator forced expiratory volume in one second (FEV_1_ % predicted), also scored from 0 to 6. The final score is the average of all seven items, with values <0.75 indicating controlled asthma and values ≥1.5 indicating uncontrolled asthma.^34^

Fidelity monitoring and interview with health professionals: During the administration of the COLA-6 questionnaire, the researcher will observe the beginning and at the end of all applications by the healthcare professional to evaluate fidelity in the application process (MARES-2). The researcher will observe the application technique of questions and peak flow, total application time, accuracy of scoring and whether the educational leaflet was correctly provided. If any inconsistency or error is identified in the first application, the researcher will intervene and provide guidance for correction in subsequent assessments.^44^ After administering the COLA-6 to 50 patients, each healthcare professional will participate in a face-to-face, semistructured interview conducted by the UFSCar research team. These interviews aim to explore barriers and facilitators related to implementing the tool in a real-world PHC setting. Interviews will be audio-recorded, transcribed verbatim and anonymised prior to analysis. The questions (provided in the online supplemental material) will address feasibility aspects such as the time required to complete the questionnaire, difficulties encountered, clarity of the questions, uncertainties during application integration of the tool into the work routine and patient receptiveness. Additionally, data will be collected to characterise each healthcare professional, including their profession, age, sex, length of professional training and years of work at the BHU/FHU.

Interview with patients: In MARES-3, semistructured interviews will be conducted with a sample of 30 patients diagnosed with abnormal spirometry or diagnosis of CRD and high risk for CRDs previously identified in MARES-1 and MARES-2, respectively: 10 with COPD, 10 with asthma and 10 with PRISm or restriction. These face-to-face interviews will be open-ended questions, audio-recorded, transcribed and anonymised before analysis. Participants will be selected to ensure diversity in gender, age, socioeconomic status and employment background. The interview (questions included in the online supplemental material) will explore how participants accessed healthcare services for diagnostic confirmation and treatment, and how this process affected their health status. Topics will include barriers and facilitators encountered, how doubts were clarified, the type of education they received regarding the disease, diagnosis and treatment, and whether any changes occurred in their lifestyle or routine following diagnosis or treatment. Additionally, a quantitative questionnaire will be administered to all participants to collect information on healthcare access, type of treatment received, medication access and treatment adherence.

### Data management

All procedures will comply with the Brazil General Personal Data Protection Law, Law No. 13,709/2018 and UK General Data Protection Regulation. This includes adherence to standard operating procedures designed to preserve the confidentiality, integrity and availability of information. Data will initially be collected using paper case report forms (CRFs) before being entered into a REDCap database. During this process, all data will be pseudonymised to ensure security and privacy for both patients and participating healthcare professionals. The paper forms, which contain patient identifiers, will be securely stored in a locked file cabinet, separate from other data collections, in a locked room at the Cardiopulmonary Physiotherapy Laboratory of the Department of Physiotherapy at UFSCar, under the responsibility of the Brazil-based principal investigator.

Data management and curation will be overseen by the lead researcher at UFSCar (RGM), the project manager (VC-S) and the postdoctoral fellow at UFSCar (NTL). After 10 years, all paper forms will be destroyed by shredding. Deidentified datasets required for reproducing the prespecified analyses will be made available via Research Electronic Data Capture (REDCap) to both the UFSCar and UCL research teams. Electronic CRFs will be stored on REDCap, a Good Clinical Practice (GCP) compliant database used by UCL. Additionally, the entire UFSCar research team will receive comprehensive training on data collection and storage procedures, as well as training in good research practices.

### Statistical methods

#### Sample size

MARES-1: The sample size calculation was based on the performance of the COLA-6 questionnaire for diagnosing COPD in the GECo study (sensitivity: 45.7%; specificity: 86.5%) and assuming a COPD prevalence of 17% in Brazil.^45^ With these assumed values, a minimum sample size of N=859 individuals will allow us to estimate the area under the ROC curve (AUC) to within 4.5% (based on a 95% CI). According to prevalence studies for CRDs in Brazil, we calculate that we will identify approximately 146 individuals with COPD, 77 with asthma and 86 with Preserved Ratio Impaired Spirometry (PRISm) or restriction in this study, giving an estimated sample of 309 with abnormal spirometry and 550 with normal spirometry. We will case-find in more people if the expected prevalence is lower to reach these targets.

MARES-2: The number of BHU/FHU that will be involved in this study, as well as the number of health professionals (N=20) responsible for applying the COLA-6 questionnaire, was a pragmatic decision. Based on the GECo study,^19^ of the total individuals evaluated (N=1000), we anticipate that 16.4% (N=164) will be identified as at high risk for CRDs (COLA-6≥4) and 836 will be identified as at low risk for CRDs. We will case-find in more people if the expected prevalence is lower to reach these targets.

MARES-3: The sample size expected (N=473) is dependent on samples obtained from MARES-1 and MARES-2. Considering the prevalence found in the GECo study^19^ and prevalence available for the Brazilian population, we estimate that we will have up to 218 individuals with COPD, 100 with asthma and 100 with PRISm.

### Data analysis

MARES-1: We will summarise the demographic and clinical characteristics of participants overall. Based on spirometry results, we will report the prevalence of COPD, asthma, PRISm and restriction, alone and in combination, with 95% CIs. For the primary analysis, we will calculate the AUC of the ROC curves for COLA-6 compared with the gold standard spirometry diagnosis separately for combined CRDs, asthma, COPD, PRISm and restriction. Using the previously published threshold for COLA-6 scores of ≥4,^19^ we will also calculate sensitivity, specificity, and negative and positive predictive values. In addition, based on the ROC curve, we will identify the COLA-6 score threshold that achieves sensitivity of at least 90%. Similar AUC analyses will be conducted for SBQ and results compared with those for COLA-6.

MARES-2: Descriptive statistics will be used to characterise the patient and healthcare professional demographics. Quantitative analysis will assess fidelity data, with categorical variables reported as frequencies (percentages). The qualitative analysis of the interviews will follow Braun and Clarke,^46^ which consists of six phases: (1) familiarisation with the data set; (2) coding; (3) generation of initial themes; (4) development and review of themes; (5) refinement, definition and naming of themes and (6) writing. The initial analysis of the transcripts obtained from the interviews will be carried out using the Web Qualitative Data Analysis software (University of Aveiro, Aveiro, Portugal) and completed manually using a Microsoft Excel spreadsheet. To ensure reliability, the criteria of credibility, transferability, dependability and confirmability will be used.^47^

MARES-3: Descriptive statistics will be used to describe the individual samples from MARES-1 and MARES-2. For patients from MARES-2, the proportions diagnosed with CRDs at spirometry will be calculated. For individuals in MARES-1, who have longitudinal data, the mean change in spirometric values from baseline to 6 months will be summarised and the proportion with improved values will be reported. Additionally, baseline and 6-month CAAT and ACQ-7 questionnaire scores, and FeNO will be summarised and mean changes reported. Estimates will be provided with 95% CIs. The qualitative analysis of the interviews will follow Braun and Clarke,^46^ as described above for MARES-2.

### Patient and public involvement

PPI is integral to our research, ensuring its relevance and impact in real-world settings. Nine community members from São Carlos and São Paulo, living with CRDs and NCDs, who attend PHC services, along with three healthcare professionals involved in PHC, have been engaged since the initial phase of the research project and will remain involved throughout its duration. To guarantee comprehensive and meaningful participation, community members were involved from the project’s inception through to its conclusion, with the frequency and timing of meetings aligned with the project timeline. Accordingly, PPI member participation was structured into four stages: Stage 1: Priority setting and proposal development; Stage 2: Review of research design and dissemination, assisting with the preparation of educational materials for study participants and guiding recruitment strategies; Stage 3: Annual monitoring, developing approaches to maintain participant involvement and producing periodic project updates; Stage 4: Research evaluation, collaborating on the creation of information for the local community, verifying that members understand and interpret the results consistently, and demonstrating the relevance and practical application of the study findings in patients’ lives.

### Ethics and dissemination

Full ethical approval was obtained from the Ethics Committee of the UFSCar and UNISA (reference number: MARES-1: 85805425.4.1001.5504; MARES-2: 85837225.7.1001.5504; MARES-3: 85813325.3.1001.5504) and UCL (2024/0048). Informed consent will be obtained from each participant. For dissemination, the research team will prepare a final article for submission to peer-reviewed academic journals and present the findings at national and international conferences.

## Supplementary material

10.1136/bmjopen-2025-109950 online supplemental file 1
