# Supplementary material for: Mitigating chronic respiratory disease through the lens of multimorbidity: the MARES mixed-methods study protocol
Source: BMJ Open. 2026 Jan 16;16(1):e109950. doi: 10.1136/bmjopen-2025-109950 (PMC12815162; doi:10.1136/bmjopen-2025-109950)
Supplement: online supplemental file 1 [file bmjopen-16-1-s001.docx]

**Supplementary Material**

**Title: Mitigating Chronic Respiratory Disease through the lens of Multi-Morbidity: The MARES mixed-methods study protocol**

**Authors**: Renata Gonçalves Mendes^1^, Naiara Tais Leonardi^1^, Viviane Castello-Simões^1^, Débora Mayumi de Oliveira Kawakami^1^, João Victor Rolim de Souza^1,2^, Nathany Souza Schafauser-Segundo^1^, Rodrigo Polaquini Simões^1^, Fernanda Gabriely Pinto^1^, Gustavo Henrique Guimarães Araujo^1^, Marcela Maria Carvalho da Silva^2,3^, Cristiane Shinohara Moriguchi^1^, Francisco José Barbosa Zörrer Franco^4^, Valéria Amorim Pires Di Lorenzo^1^, Rachael E Jordan^5^, Sonia Maria Martins^6^, [Trishul Siddharthan](https://pubmed.ncbi.nlm.nih.gov/?term=Siddharthan+T&cauthor_id=33173289)^7^, Saleh Al Sharmah^8^, Julie A Barber^9^, John R Hurst^8^

**Affiliation**^: 1^Department of Physical Therapy, Federal University of Sao Carlos, SP, Brazil; ^2^Santo Amaro University, SP, Brazil; ^3^Bioengineering Program, Scientific and Technological Institute, Brasil University, SP, Brazil; ^4^Rede D’Or - Hospital Sao Luiz, SP, Brazil; ^5^Department of Applied Health Sciences, College of Medicine and Health, University of Birmingham, BHAM, United Kingdom; ^6^Center for Public Health Studies (CESCO) at the University Center of the ABC Medical School, SP, Brazil; ^7^Department of Medicine, Division of Pulmonary, Critical Care and Sleep Medicine, University of Miami, Miller School of Medicine, FL, United States; ^8^UCL Respiratory, University College London, LD, United Kingdom; ^9^Department of Statistical Science, University College London, LD, United Kingdom.

**S1. COLA-6 questionnaire - Portuguese**

This is the COLA questionnaire, translated and cross-culturally adapted into Brazilian Portuguese by Kabbach et al, 2023. The questionnaire consists of three parts: (1) six questions addressing respiratory symptoms, functional status, and exposure to risk factors; (2) one question related to age; and (3) the peak expiratory flow (PEF) value. Patients receive 1 point for each affirmative response to the seven questions, 1 point if they are aged ≥55 years, 1 point if their PEF is between 250–399 L/min, and 2 points if their PEF is <250 L/min, with a range from 0 to 9 points.

**
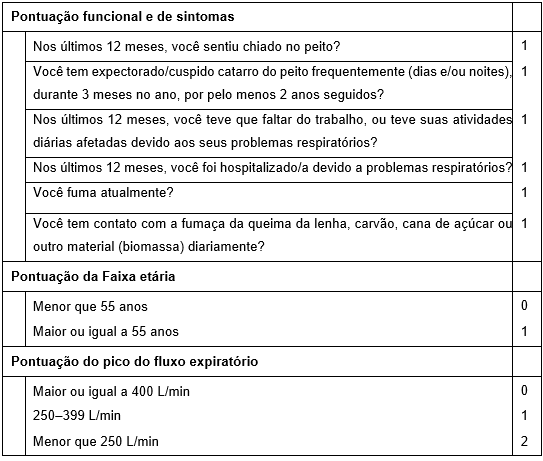
**

Kabbach EZ, Leonardi NT, Siddharthan T, Borghi-Silva A, Alqahtani JS, Hurst JR, et al. Case-Finding tool for COPD in LMIC (COLA) - translation and cross-cultural adaptation into Brazilian Portuguese language. Rev Saude Publica [Internet]. 2023 Sep 14;57(1):63. Available from: https://www.revistas.usp.br/rsp/article/view/217383

**S2. Interview with health professionals**

We would like to understand the barriers and facilitators in implementing the COLA questionnaire in your routine care. For this, we will ask you some questions, and you will be able to respond freely. This interview will be recorded.

1. 1. What is the average time required to administer the questionnaire? [ ][ ] minutes.
2. Do you consider this administration time to be short, acceptable, or long, considering its potential implementation into your primary care routine?
3. What difficulties did you encounter while administering the questionnaire?
4. Did you have any doubts or uncertainties while administering the questionnaire?
5. Were there any refusals from patients to complete the questionnaire? If yes, would you consider the refusal rate low, moderate, or high? What were the reasons given?
6. Did patients have any difficulties understanding the questions?
7. Did patients have any difficulties performing the peak expiratory flow test?
8. How well did the questionnaire integrate into your routine care at Primary Health Care?
9. Does it make sense to you to administer this questionnaire only to people with a long-term condition? Why or why not?
10. Would you use the COLA questionnaire in your routine care? Please explain why.
11. Do you believe administering the COLA questionnaire could help facilitate the diagnosis of chronic respiratory diseases?
12. What is your opinion on the COLA questionnaire?
13. Is there anything else you would like to share regarding your experience with administering the COLA questionnaire?

**S3. Interview with patients**

I will ask you some questions regarding your experience after our last contact (6 months ago).

*Patients- MARES-1*

1. Six months ago, we conducted a spirometry test and discussed your diagnosis of *COPD, asthma, or abnormal spirometry*. Can you tell us what you thought about it at the time? How did you feel, and what questions or concerns did you have?
2. What did you understand about your spirometry test results?
3. After receiving your diagnosis, did you seek more information about this lung disease? If so, what sources did you use, and what was your experience like?
4. Did you seek respiratory medical care after receiving your spirometry test results? If so, can you tell us about your experience? Did the doctor request any additional tests? If not, could you share why you did not seek respiratory medical care?
5. (For patients who had a medical consultation) After seeing a doctor, did you start any treatment? If yes, what treatments were prescribed, and what has your experience been like? If not, could you explain why? Have you faced any difficulties with treatment?
6. Has anything changed in your daily life or routine since our last contact six months ago? Please share your experience after receiving the diagnosis of *COPD, asthma, or abnormal spirometry*.

*Patients- MARES-2*

1. Six months ago, we conducted a questionnaire and discussed your risk of having a lung disease. Can you share what you thought about it at the time? How did you feel, and what questions or concerns did you have?
2. What did you understand about your questionnaire results?
3. Since receiving your results, did you try to learn more about lung disease? If so, what sources did you use, and what was your experience like?
4. Did you seek respiratory medical care after receiving these results? If so, can you tell us about your experience? Did the doctor request any additional tests? If not, could you share why you did not seek respiratory medical care?
5. (For patients who had a medical consultation) After seeing a doctor, did you start any treatment? If yes, what treatments were prescribed, and what has your experience been like? If not, could you explain why? Have you faced any difficulties with treatment?
6. Has anything changed in your daily life or routine since our last contact six months ago? Please share your experience since we discussed your risk of developing a respiratory disease.
