# Supplementary material for: Mitigating chronic respiratory disease through the lens of multimorbidity: the MARES mixed-methods study protocol
Source: BMJ Open. 2026 Jan 16;16(1):e109950. doi: 10.1136/bmjopen-2025-109950 (PMC12815162; doi:10.1136/bmjopen-2025-109950)
Supplement: Supplementary file 2 [file bmjopen-2025-109950.reviewer_comments.pdf]

PEER REVIEW HISTORY

BMJ Open publishes all reviews undertaken for accepted manuscripts. Reviewers are asked to complete a checklist review form and are provided with free text boxes to elaborate on their assessment. These free text comments are reproduced below.

ARTICLE DETAILS

Title (Provisional)

Mitigating Chronic Respiratory Disease through the lens of Multi-Morbidity: The MARES mixed-methods study protocol

Authors

Mendes, Renata Gonçalves; Leonardi, Naiara Tais; Castello-Simões, Viviane; Kawakami, Débora Mayumi Oliveira; Souza, João Victor Rolim; Schafauser-Segundo, Nathany Souza; Simões, Rodrigo Polaquini; Pinto, Fernanda Gabriely; Araujo, Gustavo Henrique Guimarães; Silva, Marcela Maria Carvalho; Moriguchi, Cristiane Shinohara; Franco, Francisco José Barbosa Zörrer; Pires Di Lorenzo , Valéria A; Jordan, Rachel; Al Sharmah, Saleh; Martins, Sonia; Siddharthan, Trishul; Barber, Julie; Hurst, John R.

VERSION 1 - REVIEW

**Reviewer**	**1**
**Name**	**Sibomana, Jean-Pierre**
**Affiliation**	**Centre Hospitalier Universitaire de Butare, Medicine**
**Date**	**27-Oct-2025**
**COI**	**None**

It is a great protocol with an expected outcome of interest in respiratory medicine

There are some points to be addressed before being accepted.

1. MARES I: a sample of 859 participants will take COLA 6 and same group undergo Spirometry, CAAT, ACQQ7 and FENO? Did you predict how long one patient will stay with the researcher and assess if there is a concern? Please state the grade of quality of spirometry you will use for interpretation and comparison.

What will you do for participants with poor spirometry performance for a statistical analysis ie the sample predicted to be analysed for statistical significance.

Can you clarify the cut of 30 years for participation? CRD especially asthma and NCD are common in young as well!

In the methodology, it would be advised to mention how research team were adequately trained for getting appropriate data

MARES III: Follow up time for 6 month, please mention possible confounders which might introduce biases like difference in health care resources utilization.

You did not mention what you will do to maintain the participation and what you will do for those lost to follow up.

VERSION 1 - AUTHOR RESPONSE

Reviewer: 1
Dr. Jean-Pierre Sibomana, Centre Hospitalier Universitaire de Butare.

Comments to the Author:
It is a great protocol with an expected outcome of interest in respiratory medicine There are some points to be addressed before being accepted.
Thank you, Dr. Sibomana, for reviewing our paper and for your valuable contributions.

1. MARES I: a sample of 859 participants will take COLA 6 and same group undergo Spirometry, CAAT, ACQQ7 and FENO? Did you predict how long one patient will stay with the researcher and assess if there is a concern?
Autor's response: Thank you for your comment. In MARES 1, we will collect these measures in all 859 participants at one visit. We conducted a pilot, and the entire assessment took around one and a half hours. We have found this acceptable to participants. The assessments are scheduled for a time in agreement by the participants, and they are informed in advance about the estimated duration. We also have a team of trained clinical researchers to ensure that we reach the planned sample size.

Please state the grade of quality of spirometry you will use for interpretation and comparison.
Autor's response: Thank you for this good suggestion. As mentioned in the manuscript, spirometry will follow American Thoracic Society and European Respiratory Society standards. We will use A or B quality grades for interpretation and comparison. We have included this information in the manuscript for clarity (Methods, Assessment, Spirometry, page 12): 'Testing will follow American Thoracic Society and European Respiratory Society standards, ensuring A or B quality grades'.

What will you do for participants with poor spirometry performance for a statistical analysis ie the sample predicted to be analysed for statistical significance.

Autor's response: Thank you for raising this important question. Participants with unacceptable spirometry performance, based on ATS/ERS quality criteria, will be excluded from spirometry-related analyses. To ensure adequate statistical power and the validity of our findings, we will continue data collection until the target sample of 859 participants with acceptable-quality spirometry (A–B grades) is reached. All exclusions and their reasons will be documented in a flow diagram to maintain transparency and allow assessment of potential selection bias.

Can you clarify the cut of 30 years for participation? CRD especially asthma and NCD are common in young as well!
Autor's response: Thank you for this comment. We agree that CRD, especially asthma, and NCDs can also be common in younger adults. However, our target population is

specifically designed to assess multi-morbidity and all available data suggests that this becomes more common with increasing age. For this reason, we defined the ≥30 years cut-off for participation.

In the methodology, it would be advised to mention how research team were adequately trained for getting appropriate data.

Autor's response: Thank you for your suggestion. From the beginning of the project, the research team have been involved in all steps of the process, including protocol development and CRF design. The training included meetings and practical workshops covering all the assessments, aiming to standardize procedures and ensure quality. In total, the team completed five full days of training (8 hours per day), totalling 40 hours. We have added a sentence to the manuscript for clarity (Methods, Assessments, page 11): 'All assessments will be conducted by a team of clinical researchers who were previously trained to ensure the accuracy and consistency of data collection.'

2. MARES III: Follow up time for 6 months, please mention possible confounders which might introduce biases like difference in health care resources utilization.

Autor's response: Thank you for this observation. This is an important aspect. As mentioned in the manuscript, we will collect detailed personal information, including age, sex, ethnicity, education and socio-economic status. We will also collect comprehensive data on all types of healthcare utilization, medications, differences in access, and available resources. These variables will be included in quantitative analyses to allow triangulation with qualitative findings. Considering these potential confounders is essential to understanding differences in access to healthcare services, as well as the barriers and facilitators influencing healthcare utilization. We have now included a sentence in the Methods section (Assessment, Interview with patients, page 15): 'Additionally, a quantitative questionnaire will be administered to all participants to collect information on healthcare access, type of treatment received, medication access, and treatment adherence'.

You did not mention what you will do to maintain the participation and what you will do for those lost to follow up.

Autor's response: Thank you for raising this concern. We will not maintain active contact with participants during the 6-month follow-up period, as this could influence the outcomes in relation to healthcare access. However, we will collect multiple contact options at baseline and will make up to three attempts to reach participants by telephone at follow-up. We acknowledge that loss to follow-up is itself an informative outcome, and participants who cannot be reached will be described and analysed accordingly. We included a sentence in the manuscript (Method, Data collection, measures, management, and analysis; MARES 3, Page 10): 'We will collect multiple contact options at baseline and will make up to three telephone attempts at follow-up; participants lost to follow-up will be documented and analysed'.

VERSION 2 - REVIEW

**Reviewer**	**1**
**Name**	**Sibomana, Jean-Pierre**
**Affiliation**	**Centre Hospitalier Universitaire de Butare, Medicine**

Date **01-Dec-2025**

COI

All concerns highlighted have been addressed appropriately
